# Epilepsy, seizures and hyperexcitability—a challenge in neurology

**DOI:** 10.1186/s42466-025-00445-5

**Published:** 2025-11-04

**Authors:** Felix Rosenow, Peter Berlit, Yvonne Weber

**Affiliations:** 1 Goethe University Frankfurt, Epilepsy Center Frankfurt Rhine-Main, Department of Neurology, Center of Neurology and Neurosurgery, University Medical Center, Frankfurt, Germany; 2 Center for Personalized Translational Epilepsy Research (CePTER), Goethe University Frankfurt, Frankfurt, Germany; 3 German Neurological Society, Berlin, Germany; 4 Department of Epileptology and Neurology, RWTH Aachen University, Aachen, Germany

Epileptic seizures are a frequent and often initial symptom and relevant complication of a wide range of brain disorders. They indicate cortical involvement and the analysis of seizure evolution contains lateralizing and localizing information for the observant clinician.

By definition, acute symptomatic seizures occur in the acute phase of CNS disorders, usually within 7 days of onset and have negative impact on the outcome. Seizures usually spontaneously stop within 1–3 min. If seizure termination mechanisms fail and seizures continue for > 5–10 min status epilepticus has occurred, a neurological emergency associated with a case fatality rate of about 25%.

Finally, epilepsy, defined by spontaneously recurring seizures, is one of the most frequent chronic neurological conditions with a prevalence of 0.6–0.7% of the population, most frequent in the very young and the very old. Epilepsy in associated with a 2.5-fold increase in mortality, frequent injuries and a reduction in quality of life, social and economic participation.

For these reasons the German Society for Neurology (www.dgn.org) has chosen “Epileptic seizures—a neurological challenge” as main topic of its annual meeting in November 2025. Besides several sessions dedicated to epilepsy and epileptic seizures the DGN-congress will put emphasis on the impact of hyperexcitability as therapeutic target in the management of other neurological disorders such as brain tumors, neuroimmunological diseases including autoimmune encephalitis and multiple sclerosis, stroke and neurodegenerative disorders. Furthermore, differential diagnoses including syncope and dissociative seizures will be highlighted.

Accompanying the annual congress of the DGN three exemplary review articles will be published in this the October and November issues of Neurological Research and Practice (NRAP) providing state of the art discussions [5, 7, 22] 

Dive et al. [5] explore recent insights into the epidemiology, clinical impact, pathophysiology, and therapeutic strategies for glioma associated epilepsy (GAE). They consider epilepsy in three main groups. A) Low grade tumors harbouring IDH-mutations, high grade tumors—mainly glioblastomas—and low-grade epilepsy-associated tumors (LEAT), a distinct group with a high risk of seizures and frequently treated by epilepsy surgery. The pathomechanisms of GAE are reviewed with regard to tumor metabolism, neuroinflammation [6, 10], increased glutamatergic activity, as well as the interaction between neoplastic and non-neoplastic cells [18]. The roles of IDH and mTOR signaling pathways in tumor growth and epileptogenesis and the emerging role of therapeutic approaches targeting these pathway and related somatic mutations are put into focus. The authors consider how integrating molecular profiling—particularly assessment of DYRK1A, EGFR mutations and mTOR activation—could inform personalized treatment strategies [3]. Data on pros and cons of the most frequently used anti-seizure medications (ASM) in GAE, as well as the therapeutic potential of perampanel to modulate the neuron-glioma synaptic network and reduce seizure frequency currently under investigation [9] are reviewed.

Willems et al. review nonconvulsive status epilepticus (NCSE) as an underdiagnosed cause of persistent unconsciousness in critically ill patients found in 8–37% of patients with unexplained coma, when applying continuous EEG (cEEG) [4, 16]. NCSE predicts an increased mortality and poorer long-term outcomes [11]. Outcome can be predicted using different scores such as STESS, EMSE and—NCSE-specific—the SACE sore [12]. The need and diagnostic gain of high ressource cEEG versus intermittent EEGs—often used in Germany—is discussed, and methods to estimate the recording time needed depending on initial EEG findings and the clinical syndrome such as the 2HELPS2B score [20] are described. Current treatment strategies [2] and novel long term outcome prediction tools such as the ACD score incorporating age at onset, level of consciousness at admission, and duration of SE are detailed [15].

Heckelmann et al. [7] review our current knowledge regarding the management of epilepsy in women of childbearing potential and during pregnancy and breast feeding. Treatment guidelines are based on solid evidence from clinical trials and pregnancy registries such as EURAP (https://eurapinternational.org). We have learned which ASM to use and which to avoid [8] and that rates of major malformations have been decreased from 6.1 to 3.7% over the last two decades based on these data [1]. It is now widely recommended to use 400–800 µg of folic acid as prophylaxis in WwE of childbearing age and breastfeeding is encouraged. Monitoring ASM levels and adjusting doses has been suggested to prevent breakthrough seizures during pregnancy [13].

The 2025 DGN-congress also provides an overview and in depth discussion of the role of seizures in the prognosis and clinical course of different neurological disorders including stroke where epilepsy lead to a doubling of case fatality rates [19] and multiple sclerosis in which it predicts poor cognitive outcome [14]. During the presidential symposium the role of hyperexcitability in the pathophysiology and treatment of dementias are dissected, to answer the question if seizures are just an epiphenomenon or indispensable to understand the pathophysiology and find valid treatment targets [17, 21].

Novel treatment strategies and treatment targets in the epilepsies will be at the heart of the epilepsy specific symposia. These include personalized treatment approaches for monogenetic epilepsies by ASO-therapies as well as stereotactic laser thermo-ablation (LITT) as surgical treatment option just arriving in Europe. Together with representatives from pharmaceutical industry the future needs and developments in drug treatment will be considered, and how drugs can be guided to the epileptic tissue in focal epilepsy. On the last day of the meeting patient mobility with regards to driving ability, a major concern for many of our patients, and the role of autonomous driving in this regard will be put into focus.

Taken together, the annual DGN-congress in Berlin, Germany on November 12th-15th and the reviews in this issue of NRP provide a detailed, concise overview and plenty of thought-provoking new data and ideas regarding epilepsy and epileptic seizures as one of the many challenges in current neurology.

You are cordially invited to read and participate!
